# Nitrogen-Polar (000$\bar{1}$) GaN Grown on c-Plane Sapphire with a High-Temperature AlN Buffer

**DOI:** 10.3390/ma10030252

**Published:** 2017-03-02

**Authors:** Jie Song, Jung Han

**Affiliations:** 1 Department of Electrical Engineering, Yale University, New Haven, CT 06520, USA; jung.han@yale.edu; 2 Saphlux Inc., Branford, CT 06405, USA

**Keywords:** MOCVD, N-polar, GaN, AlN buffer

## Abstract

We demonstrate growing nitrogen-polar (N-polar) GaN epilayer on c-plane sapphire using a thin AlN buffer layer by metalorganic chemical vapor deposition. We have studied the influence of the AlN buffer layer on the polarity, crystalline quality, and surface morphology of the GaN epilayer and found that the growth temperature of the AlN buffer layer played a critical role in the growth of the GaN epilayer. The low growth temperature of the AlN buffer results in gallium-polar GaN. Even a nitridation process has been conducted. High growth temperature for an AlN buffer layer is required to achieve pure N-polarity, high crystalline quality, and smooth surface morphology for a GaN epilayer.

## 1. Introduction

Nitrogen-polar (000$\bar{1}$) (N-polar) GaN is attractive in III-nitride research because of its opposite spontaneous polarization field direction compared to gallium-polar (Ga-polar) structures. The reversal of the spontaneous polarization causes a significant change of heterojunction band profiles and device characteristics. In the past, N-polar c-plane light-emitting diodes (LEDs) have exhibited a reduced efficiency droop and a lower threshold current density [1,2], respectively, and have enhanced the incorporation of indium into InGaN multiple quantum wells (MQWs) for long wavelength visible LEDs [3,4,5]. The location of the two-dimensional electron gas in AlGaN/GaN heterojunction field-effect transistors can be altered by switching from Ga- to N-polar orientation to achieve different device applications [6,7]. The concurrent growth of GaN with Ga- and N-polarity side-by-side opens up applications in non-linear optics and acoustics [8], lateral polarity junction [9], and polarity-selective patterning of GaN [10]. The growth of N-polar semipolar GaN requires an initial inversion from Ga-polarity to N-polarity by employing an appropriate nucleation layer, either AlN or GaN. The use of low-temperature GaN as a nucleation layer substrate has been well developed and understood to achieve N-polar GaN on sapphire [11,12,13,14]. However, the growth mechanism of using an AlN nucleation layer to obtain N-polarity of a GaN epilayer is unclear.

In this study, we report on the growth of an N-polar GaN epilayer on c-plane sapphire using a thin AlN buffer layer by metalorganic chemical vapor deposition (MOCVD). We studied the influence of the AlN buffer layer on the polarity, crystalline quality, and surface morphology of the GaN epilayer and found that the growth temperature of the AlN buffer layer played an important role in achieving purely N-polar GaN with high crystalline quality and a smooth surface. A high growth temperature above 850 °C for the AlN buffer layer is required to achieve pure N-polarity, high crystalline quality, and smooth surface morphology in a GaN epilayer.

## 2. Experiment

N-polar GaN was grown on c-plane (0001) sapphire substrate with 2° off-cut towards A-axis by MOCVD. Before the growth, a nitridation process was conducted on sapphire substrates at 950 °C in a mixture of N_2_ and NH_3_. After nitridation, a 20-nm-thick AlN buffer layer was grown on the substrate, followed by a GaN epilayer grown at 1050 °C, 100 mbar, and 1 slm NH_3_, with a growth rate of about 1.2 μm/h. After growth, the polarity was ascertained by wet etching in KOH (4.5 M) solution at room temperature (RT) for 5 min. Surface morphology was examined by Nomarski optical microscopy, Hitachi SU-70 scanning electron microscopy (SEM, Tokyo, Japan), and atomic force microscopy (AFM, FastScan, BRUKER, Karlsruhe, Germany). Crystalline quality was characterized by X-ray diffraction (XRD, SmartLab, Rigaku, Tokyo, Japan).

## 3. Results and Discussion

**Figure 1:**
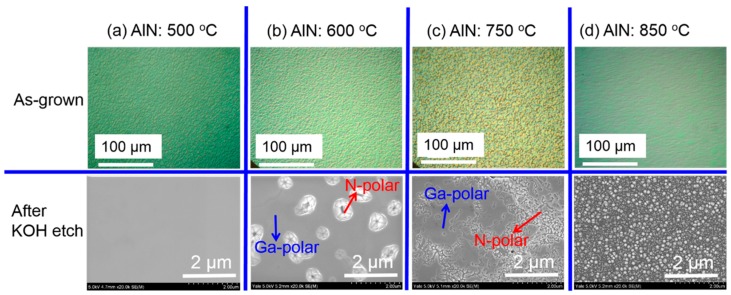
Optical microscope images of as-grown (top array) and SEM images (bottom array) of KOH etched GaN epilayers with AlN buffer layers grown at temperatures of (**a**) 500 °C; (**b**) 600 °C; (**c**) 750 °C; and (**d**) 850 °C, respectively.

First, we studied the influence of growth temperature of an AlN buffer on the properties of GaN epilayers and found that the growth temperature of the AlN buffer played a critical role in achieving purely N-polar GaN with a smooth surface and high crystalline quality. Figure 1 shows the surface morphologies of 500 nm GaN epilayers grown on c-plane sapphire substrates with an AlN buffer growth temperatures of (a) 500 °C; (b) 600 °C; (c) 750 °C; and (d) 850 °C, respectively. The top array of Figure 1 shows the optical microscopy images of the as-grown GaN epilayers and the bottom array of Figure 1 shows the SEM images of GaN after KOH etching. It is known that the Ga-polar terminated GaN is very inert to KOH etching, whereas N-polar terminated GaN would be etched by KOH solution and the surface of N-polar GaN will become very rough. As seen in Figure 1a, the surface of GaN with an AlN buffer grown at 500 °C is featureless after KOH etching, indicating that GaN with an AlN buffer grown at 500 °C is purely Ga-polar. With increase the AlN buffer growth temperature to 600 °C, it shows that the majority of the area is still Ga-polar with the unetched surface after KOH etching (marked by a blue arrow in Figure 1b). However, N-polar GaN starts to form with etching holes appeared after KOH etching, marked by a red arrow in Figure 1b. With increasing growth temperature of the AlN buffer to 750 °C, it shows that dramatically increased area of GaN has already become N-polar showing pyramidal rough surface after KOH etching, as shown by the red arrow in Figure 1c. The unetched region of Ga-polar GaN is labeled by the blue arrow in Figure 1c. It reveals that the GaN epilayer with an AlN buffer grown at 600 to 750 °C is mixed-polar. By increasing the growth temperature of the AlN buffer further to 850 °C, GaN epilayer becomes very rough with uniform pyramids formed on the surface over the entire wafer after KOH etching, as shown in Figure 1d. GaN with an AlN buffer layer grown at above 850 °C also exhibits pyramidal surface morphology over the entire wafer after KOH etching (not shown here). It indicates that the GaN epilayer has become purely N-polar with an AlN buffer grown at a temperature of above 850 °C.

**Figure 2:**
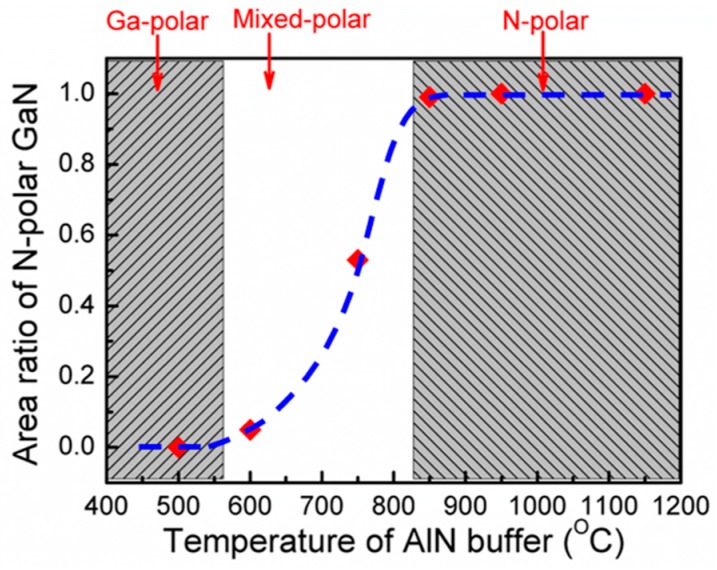
The dependence of the area ratio of N-polar on the growth temperature of the AlN buffer.

To summarize the influence of the growth temperature of the AlN buffer layer on the polarity of the GaN epilayer more quantitatively, we have calculated the area ratio of N-polarity of the GaN epilayer over the entire area and plotted the dependence of the area ratio of N-polar GaN on the growth temperature of the AlN buffer in Figure 2. As shown in Figure 2, if the AlN buffer is grown at a temperature below 600 °C, Ga-polar GaN will be formed even though an aggressive nitridation is conducted on sapphire substrate. When the growth temperature of the AlN buffer is around 750 °C, GaN with mixed-polarity will be formed. A high growth temperature of above 850 °C is required for the AlN buffer to achieve purely N-polar GaN epilayer.

At the early stage, Ito et al. studied the effect of AlN buffer deposition conditions on the properties of a GaN epilayer and realized that the NH_3_ dissociated from the growth of high-temperature AlN buffer would nitridize the sapphire and thus create an N-polar GaN epilayer [15]. Later, Liu et al. reported a Ga-polar GaN epilayer grown with a low-temperature AlN buffer on a nitridized sapphire substrate [11]. More recently, Jasinski et al. [16], Paduano et al. [17], Wu et al. [18], Kirste et al. [19], and Hussey et al. [20] found that AlN epilayers grown at high temperature directly on a nitridized sapphire without an AlN buffer were dominated by an N-polar, with Al-polarity inversion domains appearing locally. Our result is consistent with these reports. In general, nitridation coverts the sapphire (Al_2_O_3_) surface to a spinel structure aluminum-oxynitride (Al_x_O_y_N_z_) by a continuous substitution of oxygen atoms in sapphire by nitrogen, and stepwise forms N-polar AlN [21]. In our understanding, since an Al–O bond is much stronger than an Al–N bond [16], we believe that a layer of Al_x_O*_y_*N*_z_* is regenerated at low growth temperature. This layer of Al_x_O*_y_*N*_z_* leads to the metal-polar AlN buffer and subsequently a Ga-polar GaN epilayer grown on metal-polar AlN buffer [22]. When an AlN buffer layer is grown at high temperature, Al_x_O*_y_*N*_z_* starts to dissociate at high temperature and convert to pure Al–N bonds with N atoms terminated on the surface under N-rich ambient, due to the higher decomposition rate of NH_3_ at high temperature [15]. An N-polar AlN buffer is then formed on this N-terminated substrate surface, and thus N-polar GaN is generated. 

**Figure 3:**
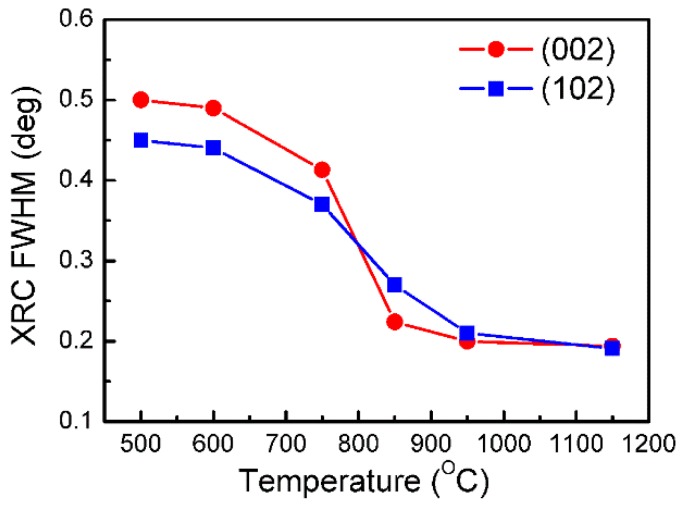
The XRC FWHMs of N-polar GaN with an AlN buffer grown at different temperatures.

The crystalline quality of the GaN epilayer with an AlN buffer layer grown at different temperatures was characterized by XRD and the full width at half maximums (FWHMs) of XRD rocking curves (RCs) are shown in Figure 3. It is found that the crystalline quality of the GaN epilayer with an AlN buffer grown at temperature of below 600 °C is very poor with (002) and (102) FWHMs of about 0.5° and 0.45°, respectively. By increasing the growth temperature of the AlN buffer from 600 to 850 °C, the crystalline quality of the GaN epilayer is significantly improved. However, by increasing the growth temperature of the AlN buffer above 950 °C, the FWHMs of XRD RCs remain nearly the same since purely N-polar GaN is formed.

**Figure 4:**
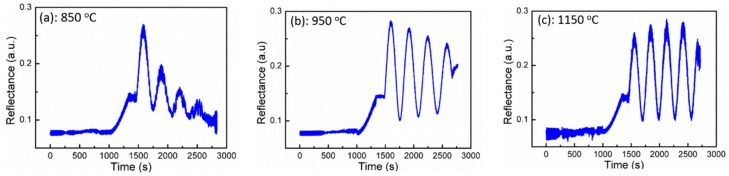
In-situ optical reflectance of N-polar GaN growth with an AlN buffer grown at temperatures of (**a**) 850 °C; (**b**) 950 °C; and (**c**) 1150 °C, respectively.

**Figure 5:**
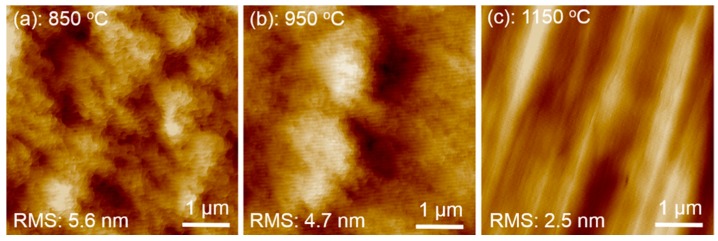
AFM images of N-polar GaN epilayers with AlN buffer layers grown at temperature of (**a**) 850 °C; (**b**) 950 °C; and (**c**) 1150 °C, respectively, with a scanning area of 5 × 5 μm^2^.

Even though the purely N-polar GaN was achieved with an AlN buffer grown at a temperature of above 850 °C, we observed a significant difference in the optical reflectance that monitors the growth evolution. The wavelength of light source used for in-situ reflectance measurement was 550 nm. Figure 4 shows the growth reflectance of GaN with AlN buffers grown at (a) 850 °C; (b) 950 °C; and (c) 1150 °C, respectively. An instant full amplitude oscillation in reflectance implying a quasi-2D growth mode is observed, indicating that these three GaN epilayers with AlN grown at 850, 950, and 1150 °C are all N-polar [13]. In addition, we observe that the reflectance trace of N-polar GaN with an AlN buffer grown at 850 °C decays very dramatically in the oscillation intensity, indicating a substantial and accumulated surface roughening. The reflectance of GaN with an AlN buffer grown at 950 °C decays much less in comparison with an AlN buffer grown at 850 °C, indicating that the surface morphology of GaN with an AlN buffer grown at 950 °C is an improvement. However, the reflectance trace of N-polar GaN with an AlN buffer grown at 1150 °C exhibits a gradual increment for the first three oscillations and then keeps constantly, indicating that a very smooth surface of GaN has been achieved. The surface morphology of N-polar GaN with AlN grown at different temperatures was further examined by AFM. Figure 5 shows AFM images of GaN grown at (a) 850 °C; (b) 950 °C; and (c) 1150 °C, respectively, with the scanning area of 5 × 5 μm^2^. The root mean square (RMS) roughnesses of the GaN epilayers extracted from AFM results are 5.6, 4.7, and 2.5 nm, respectively, for GaN with an AlN buffer grown at 850, 950, and 1150 °C. This indicates that the surface of the GaN epilayer becomes smoother by increasing the growth temperature of the AlN buffer, consistent with the results shown in Figure 4.

**Figure 6:**
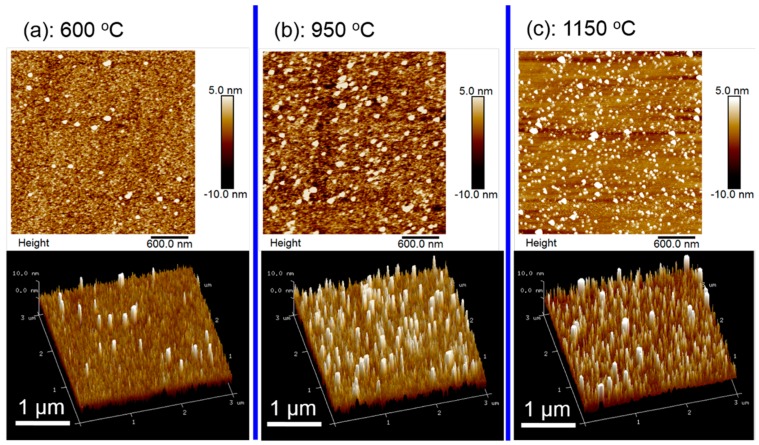
AFM images of AlN buffers grown at temperatures of (**a**) 600 °C; (**b**) 950 °C; and (**c**) 1150 °C, respectively, with a scanning area of 3 × 3 μm^2^. Upper and lower arrays show the two-dimensional and three-dimensional images, respectively.

To understand the influence of an AlN buffer on the crystalline quality of GaN epilayers, three AlN buffer layers were grown at different temperatures with growth stopped after thermal annealing and right before the growth of a high-temperature GaN epilayer. The surface morphology of the AlN buffer layer was examined by AFM. Figure 6a–c show the AFM images of the AlN buffer grown at temperatures of 600, 950, and 1150 °C, respectively. The upper and lower array show the two-dimensional and three-dimensional AFM images, respectively. The RMS extracted from AFM images are 1.4, 1.9, and 2.0 nm, respectively. This clearly indicates that the AlN buffer grown at 600 °C exhibits a very low density of large grains (white spots in AFM images) with a size of 30 to 50 nm. By increasing the growth temperature to 900 °C, the density of large size grains increases dramatically. The densities of the large-size grains are 2.3 × 10^8^, 1.7 × 10^9^, and 1.9 × 10^9^ cm^−2^ for the AlN grown at 600, 950, and 1150 °C, respectively. The high density of the large-size grains in the AlN buffer layer will promote the lateral growth and coalescence of initial GaN islands grown on AlN grains, which leads to an increased volume of defect-free columnar domains, thus improving the crystal quality of the GaN epilayer [23,24]. That is the reason why we observed much lower XRC FWHMs for the GaN epilayer with an AlN buffer grown at 900 °C compared to that grown at 600 °C in Figure 3. However, by increasing the growth temperature of the AlN buffer further to 1150 °C, the density of the large-size grain does not exhibit any significant change in comparison with the AlN buffer grown at 900 °C. Hence, the XRC FWHMs of the GaN epilayers are nearly the same as those of AlN buffers grown at a temperature of above 900 °C.

## 4. Conclusions

We have investigated the influence of the growth temperature of the AlN buffer on the polarity, crystalline quality, and surface morphology of the GaN epilayers and found that the growth temperature of the AlN buffer played an important role. A high growth temperature above 850 °C for the AlN buffer is required to achieve purely N-polar GaN. Even for purely N-polar GaN epilayer, a higher growth temperature of the AlN buffer always results in the smoother surface morphology of the GaN epilayer probably due to the better crystalline quality of the AlN buffer grown at higher temperature. The crystalline quality of the GaN epilayer is improved significantly with increasing the growth temperature of the AlN buffer layer. It can be interpreted by the grains density of the AlN buffer. AlN buffer grown at low growth temperature exhibits a much lower density of large size grains than that of the AlN buffer grown at high temperature. A high density of large size nucleation grains promotes the lateral growth and coalescence of initial GaN islands, which leads to an increased volume of defect-free columnar domains, thus improving the crystal quality of the GaN epilayer.

## References

[1] Yen S.-H., Kuo Y.-K. (2008). Polarization-dependent optical characteristics of violet InGaN laser diodes. J. Appl. Phys..

[2] Akyol F., Nath D.N., Krishnamoorthy S., Park P.S., Rajan S. (2012). Suppression of electron overflow and efficiency droop in N-polar GaN green light emitting diodes. Appl. Phys. Lett..

[3] Keller S., Fichtenbaum N.A., Furukawa M., Speck J.S., Denbaars S.P., Mishra U.K. (2007). Growth and characterization of N-polar InGaN/GaN multiquantum wells. Appl. Phys. Lett..

[4] Masui H., Keller S., Fellows N., Fichtenbaum N.A., Furukawa M., Nakamura S., Mishra U.K., DenBaars S.P. (2009). Luminescence characteristics of N-Polar GaN and InGaN films grown by metal organic chemical vapor deposition. Jpn. J. Appl. Phys..

[5] Shojiki K., Tanikawa T., Choi J.-H., Kuboya S., Hanada T., Katayama R., Matsuoka T. (2015). Red to blue wavelength emission of N-polar InGaN light-emitting diodes grown by metalorganic vapor phase epitaxy. Appl. Phys. Express.

[6] Keller S., Suh C.S., Chen Z., Chu R., Rajan S., Fichtenbaum N.A., Furukawa M., DenBaars S.P., Speck J.S., Mishra U.K. (2008). Properties of N-polar AlGaN/GaN heterostructures and field effect transistors grown by metalorganic chemical vapor deposition. J. Appl. Phys..

[7] Wong M.H., Pei Y., Palacios T., Shen L., Chakraborty A., McCarthy L.S., Keller S., DenBaars S.P., Speck J.S., Mishra U.K. (2007). Low nonalloyed Ohmic contact resistance to nitride high electron mobility transistors using N-face growth. Appl. Phys. Lett..

[8] Chowdhury A., Ng H.M., Bhardwaj M., Weimann N.G. (2003). Second-harmonic generation in periodically poled GaN. Appl. Phys. Lett..

[9] Aleksov A., Collazo R., Mita S., Schlesser R., Sitar Z. (2006). Current-voltage characteristics of n/n lateral polarity junctions in GaN. Appl. Phys. Lett..

[10] Ng H.M., Parz W., Weimann N.G., Chowdhury A. (2003). Patterning GaN microstructures by polarity-selective chemical etching. Jpn. J. Appl. Phys..

[11] Liu F., Collazo R., Mita S., Sitar Z., Duscher G., Pennycook S.J. (2007). The mechanism for polarity inversion of GaN via a thin AlN layer: Direct experimental evidence. Appl. Phys. Lett..

[12] Keller S., Fichtenbaum N.A., Wu F., Brown D., Rosales A., DenBaars S.P., Speck J.S., Mishra U.K. (2007). Influence of the substrate misorientation on the properties of N-polar GaN films grown by metal organic chemical vapor deposition. J. Appl. Phys..

[13] Sun Q., Cho Y.S., Lee I.-H., Han J., Kong B.H., Cho H.K. (2008). Nitrogen-polar GaN growth evolution on c-plane sapphire. Appl. Phys. Lett..

[14] Sun Q., Suk Cho Y., Kong B.H., Koun Cho H., Shine Ko T., Yerino C.D., Lee I.-H., Han J. (2009). N-face GaN growth on c-plane sapphire by metalorganic chemical vapor deposition. J. Cryst. Growth.

[15] Ito T., Ohtsuka K., Kuwahara K., Sumiya M., Takano Y., Fuke S. (1999). Effect of AlN buffer layer deposition conditions on the properties of GaN layer. J. Cryst. Growth.

[16] Jasinski J., Liliental-Weber Z., Paduano Q.S., Weyburne D.W. (2003). Inversion domains in AlN grown on (0001) sapphire. Appl. Phys. Lett..

[17] Paduano Q., Weyburne D. (2003). Two-step process for the metalorganic chemical vapor deposition growth of high quality AlN films on sapphire. Jpn. J. Appl. Phys..

[18] Wu Y., Hanlon A., Kaeding J.F., Sharma R., Fini P.T., Nakamura S., Speck J.S. (2004). Effect of nitridation on polarity, microstructure, and morphology of AlN films. Appl. Phys. Lett..

[19] Kirste R., Mita S., Hussey L., Hoffmann M.P., Guo W., Bryan I., Bryan Z., Tweedie J., Xie J., Gerhold M. (2013). Polarity control and growth of lateral polarity structures in AlN. Appl. Phys. Lett..

[20] Hussey L., White R.M., Kirste R., Mita S., Bryan I., Guo W., Osterman K., Haidet B., Bryan Z., Bobea M. (2014). Sapphire decomposition and inversion domains in N-polar aluminum nitride. Appl. Phys. Lett..

[21] Uchida K., Watanabe A., Yano F., Kouguchi M., Tanaka T., Minagawa S. (1996). Nitridation process of sapphire substrate surface and its effect on the growth of GaN. J. Appl. Phys..

[22] Mohn S., Stolyarchuk N., Markurt T., Kirste R., Hoffmann M.P., Collazo R., Courville A., Di Felice R., Sitar Z., Vennéguès P. (2016). Polarity control in group-III nitrides beyond pragmatism. Phys. Rev. Appl..

[23] Han J., Ng T.-B., Biefeld R.M., Crawford M.H., Follstaedt D.M. (1997). The effect of H_2_ on morphology evolution during GaN metalorganic chemical vapor deposition. Appl. Phys. Lett..

[24] Zhao D.G., Zhu J.J., Liu Z.S., Zhang S.M., Yang H., Jiang D.S. (2004). Surface morphology of AlN buffer layer and its effect on GaN growthby metalorganic chemical vapor deposition. Appl. Phys. Lett..

